# Coexistence of cat scratch disease lymphadenitis and active pulmonary tuberculosis in an immunocompetent host – a case report with metagenomic diagnosis and literature review

**DOI:** 10.3389/fmed.2025.1667171

**Published:** 2025-10-09

**Authors:** Xuan Wang, Shufang Chen, Chengqing Yang

**Affiliations:** Wuhan Pulmonary Hospital, Wuhan Institute for Tuberculosis Control, Wuhan, Hubei, China

**Keywords:** cat scratch disease, pulmonary tuberculosis, granulomatous lymphadenitis, metagenomic next-generation sequencing, coinfection, *bartonella henselae*

## Abstract

Cat scratch disease (CSD), caused by *Bartonella henselae (B. henselae)*, typically presents as localized swelling of lymph nodes following a scratch or bite from a cat. It is crucial to differentiate CSD from tuberculosis (TB), particularly in regions where TB is prevalent. This report describes a 56-year-old man who exhibited bilateral swelling of the cervical lymph nodes. Initially, he was suspected to have tuberculous lymphadenitis due to the granulomatous changes observed in a biopsy of the lymph nodes, typical signs of TB on a chest CT scan, and a positive result from an interferon-gamma release assay (IGRA). He was subsequently referred to our hospital for TB treatment. Testing of bronchoalveolar lavage fluid confirmed the presence of TB-DNA, indicating active pulmonary tuberculosis (PTB). However, further investigation revealed recent cat contact. This led to the identification of a *B. henselae* infection using metagenomic pathogen detection workflow (MetaPath™) on formalin-fixed paraffin-embedded (FFPE) histopathological sections from a cervical lymph node specimen obtained at an external hospital, which confirmed the diagnosis of CSD and ruled out TB. Through a review of the literature, we found that this represents the first documented case of concurrent active PTB and CSD-related lymphadenitis in an immunocompetent individual. It highlights the diagnostic challenges in distinguishing CSD from TB in cases of granulomatous lymphadenitis and emphasizes the need to consider CSD in patients with a history of cat exposure, showcasing the pivotal role of advanced metagenomic diagnostics in accurately diagnosing CSD.

## Background

Cat scratch disease (CSD) is an infectious disease caused by *B. henselae (B. henselae)* (1), which belongs to the gram-negative category and is part of the Proteobacteria phylum (2). Cats Proteobacteria are the primary carriers of this pathogen, which can be transmitted to humans through scratches, bites, or licks, especially from infected young cats, with a higher prevalence in autumn and winter. CSD typically manifests as localized skin lesions and swollen lymph nodes (3). In regions endemic to tuberculosis (TB), granulomatous lymphadenitis is frequently attributed to Mycobacterium tuberculosis (MTB) (4, 5), followed by atypical mycobacterial infections, fungal infections, parasitic infections, CSD, lymphogranuloma venereum, and leprosy (6, 7). Due to the similarities in histopathological features between CSD and TB, CSD remains underrecognized, and patients may be misdiagnosed with lymphoproliferative disorders or TB (6).

This report presents the first documented instance of the coexistence of pulmonary tuberculosis (PTB) with CSD-associated cervical lymphadenitis. While the lymphadenitis was attributed to CSD rather than TB, the patient exhibited cervical lymphadenopathy, which inadvertently led to the detection of PTB.

## Case presentation

**FIGURE 1:**
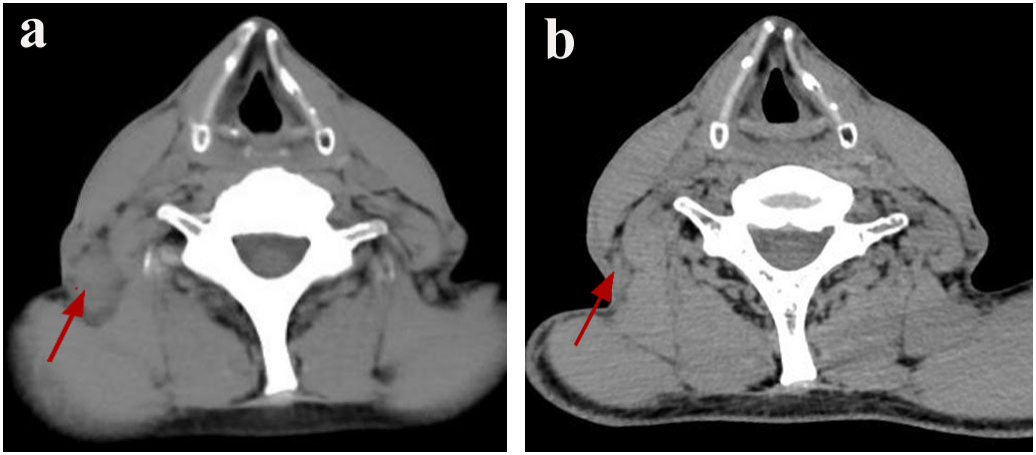
Resolution of CSD-associated lymphadenopathy on serial cervical CT. **(a)** Pre-treatment CT scan (January 8, 2025) demonstrates an enlarged right posterior cervical lymph node (red arrow). **(b)** Follow-up CT scan (March 26, 2025) shows the enlarged lymph node in the right posterior cervical space has disappeared (red arrow).

**FIGURE 2:**
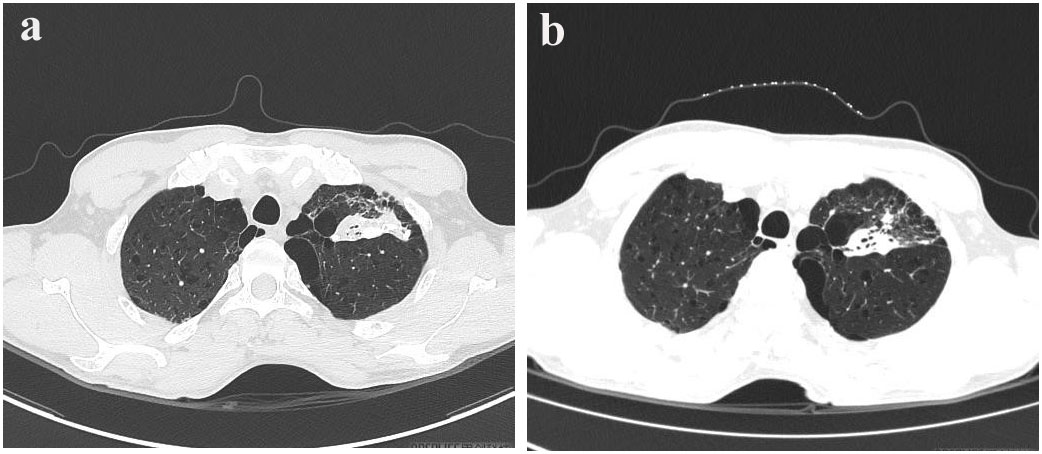
Pre- and post-treatment CT scans in pulmonary tuberculosis. **(a)** Chest CT scan on January 22, 2025. **(b)** Follow-up chest CT scan on March 26, 2025.

**FIGURE 3:**
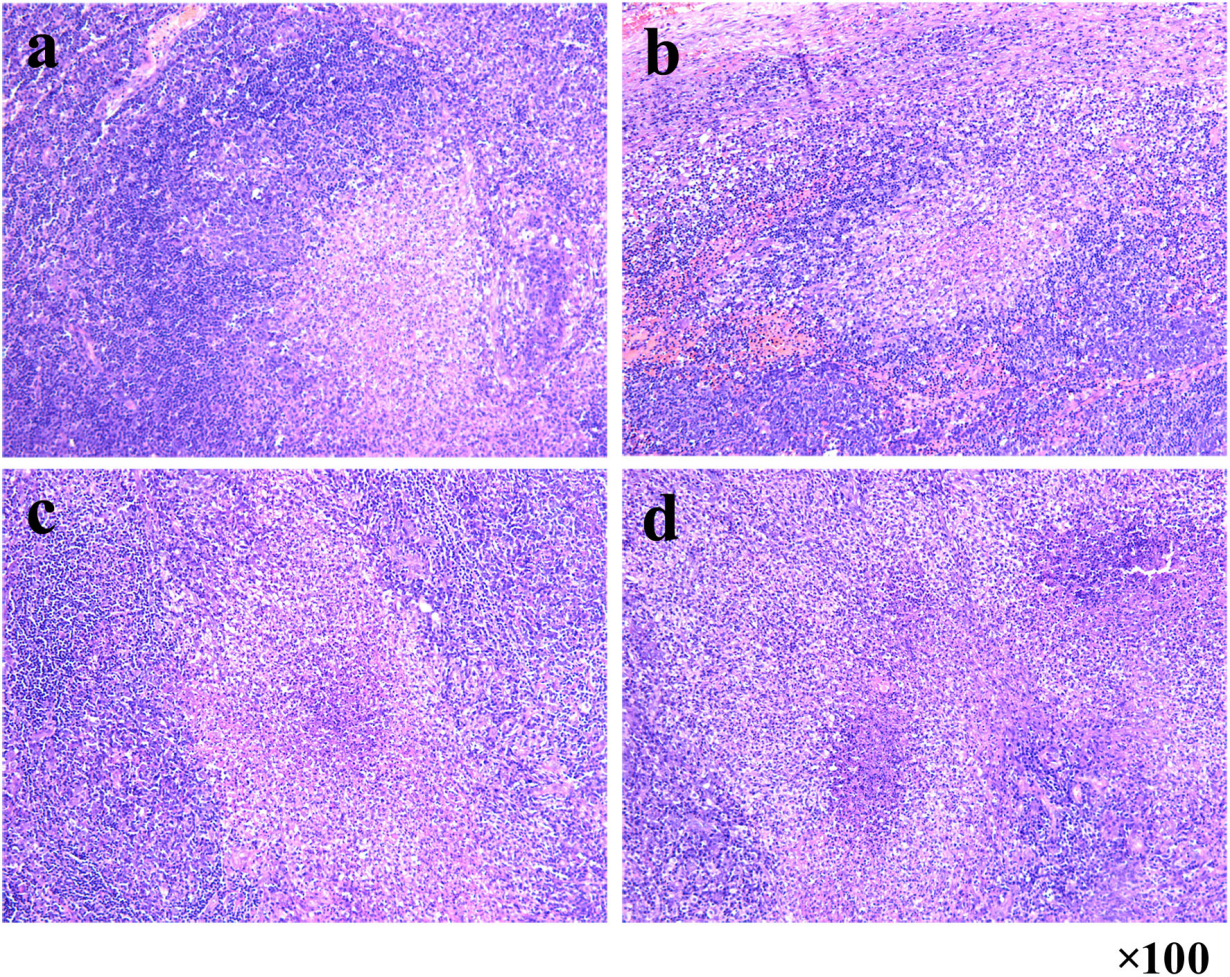
Suppurative granulomatous lymphadenitis in right cervical lymph node biopsy. **(a–d)** Microscopy reveals multiple small abscesses with central necrosis and neutrophilic infiltration, surrounded by palisaded epithelioid histiocytes (HE staining, 100 × magnification).

On January 8, 2025, a 56-year-old man presented at an external hospital with bilateral masses in his neck, having first noticed these swellings a week prior, with the right side being more prominent. The patient reported no additional symptoms such as cough, sputum production, or fever. His medical history was unremarkable for TB, diabetes, or hypertension; however, he had a 30-year history of alcohol use and smoking. Upon examination, he was alert and oriented, with no signs of jaundice or petechiae on his skin or mucous membranes. Bilateral cervical lymphadenopathy was observed, with the largest lymph node in the right posterior cervical area measuring approximately 3 cm. These nodes had intact skin, exhibited no signs of ulceration or redness, felt firm, and were non-tender. A cervical CT scan indicated bilateral lymph node enlargement (Figure 1a), while a chest CT revealed emphysema, bullae, and a calcified patch in the left upper lobe (Figure 2a). An ultrasound of the neck confirmed an increase in both the size and number of cervical lymph nodes, with the largest measuring around 2.7 × 1.0 cm on the right side. Laboratory tests, including a complete blood count, urinalysis, and assessments of liver and kidney function, yielded normal results. The patient had no significant past medical history or history of immunocompromising conditions, and T-cell subset analysis revealed normal levels of CD4 + and CD8 + cells. Serological tests for HBV, HCV, syphilis, and HIV were negative. The ESR was recorded at 11 mm/h, hs-CRP at 0.87 mg/L, PCT at 0.06 ng/mL, and CEA at 3.30 ng/mL, while the IGRA test was positive. A biopsy taken from the right cervical lymph node on January 10, 2025, revealed multiple small abscesses within the lymph node upon microscopy. The centers of these abscesses displayed necrosis accompanied by abundant neutrophilic infiltration, surrounded by epithelioid histiocytes arranged in a palisading pattern, forming suppurative granulomatous inflammation (Figures 3a–d), which raised an initial suspicion of TB. However, TB special stains, including periodic acid-Schiff (PAS), silver methenamine, and acid-fast staining of the lymph node tissue, returned negative results, and TB-DNA was not detected. On January 17, 2025, the patient was referred to our hospital with a diagnosis of pulmonary tuberculosis and cervical tuberculous lymphadenitis, attributed to his residence in a TB-endemic area, a positive IGRA result, and characteristic imaging findings of PTB.

A bronchoscopy performed at our facility revealed no irregularities; however, the bronchoalveolar lavage fluid tested positive for TB-DNA, suggesting the presence of pulmonary tuberculosis. During a detailed history review, the patient disclosed having two cats for the past 2 years. Although there was no clear record of cat scratches, the possibility of CSD was considered due to negative results from PAS, silver methenamine, and acid-fast staining, along with the patient’s exposure to cats. Metagenomic pathogen detection workflow (MetaPath™)–a probe-enriched metagenomic next-generation sequencing (mNGS) method optimized for low-biomass specimens–successfully detected *B. henselae* sequences, rather than MTB, in formalin-fixed paraffin-embedded (FFPE) histopathological sections of a cervical lymph node specimen obtained externally. MetaPath™ technology of the cervical lymph node tissue detected sequences specific to *B. henselae*, identifying 82 sequences at a relative abundance of 7.19%, thereby confirming a diagnosis of CSD. The patient presented with lymphadenopathy, and pathological findings revealed granulomatous inflammation. The differential diagnoses include infectious etiologies such as tuberculosis, non-tuberculous mycobacterial infection, and fungal infections, as well as non-infectious conditions such as sarcoidosis and lymphoma. Based on comprehensive clinical, laboratory, radiological, pathological, etiological, and molecular evaluations, the patient final diagnoses included: (1) cervical lymphadenitis related to CSD; (2) pulmonary tuberculosis; and (3) emphysema with bullae. The patient commenced anti-tuberculosis therapy on January 18, 2025, following a regimen consisting of Isoniazid, Rifampicin, Pyrazinamide, and Ethambutol for 2 months, followed by a 4-month continuation of Isoniazid and Rifampicin. Subsequently, upon the detection of *Bartonella* species via MetaPath™ testing on January 23, 2025, ciprofloxacin was added for a duration of 4 weeks to address cat-scratch disease, targeting anti-*Bartonella* therapy. A follow-up CT scan of the chest and neck conducted on March 26 indicated partial resolution of the lesion in the left upper lung (Figure 1b) and nearly complete reduction of the enlarged lymph nodes in the right posterior cervical area (Figure 2b).

## Literature review and discussion

Cat scratch disease generally resolves spontaneously in individuals with a competent immune system, initially presenting as skin lesions followed by ipsilateral regional lymphadenopathy (8). Research indicates variability in the clinical presentation of the disease. For instance, it often resolves without intervention in most children and immunocompetent individuals, while older adults or immunocompromised individuals may exhibit atypical clinical manifestations (9), including fever of unknown origin, disseminated visceral lesions, neurological complications, uveitis, bacillary angiomatosis, and infective endocarditis (10–12). The typical histopathological features of CSD in lymph nodes include granulomas with central necrosis, multinucleated giant cells, and microabscesses (6, 13–16). However, the presence of granulomatous inflammation alone does not confirm a diagnosis of CSD, as similar histological patterns can be observed in a variety of infectious and non-infectious diseases, including TB, infections from other acid-fast bacilli, fungal diseases, toxoplasmosis, lymphomas, various cancers, vasculitis, and sarcoidosis, among others (4–7).

We performed a literature review utilizing the terms “tuberculosis, cat scratch disease, granulomatous lymphadenitis” and found 15 articles that discussed case studies involving both CSD and TB. A summary comparing these cases with our current findings is presented in Table 1 (10, 11, 14–26). It is noteworthy that one case study reported the simultaneous presence of TB and CSD in the same patient (17). Matias et al. documented the onset of TB lymphadenitis 3 years after a CSD diagnosis in a patient with colon cancer who was immunocompromised (17). Bernit et al. were the first to identify a co-infection of *B. quintana* (not *B. henselae*) and TB in an HIV-positive individual suffering from generalized lymphadenitis, noting three infections occurring concurrently in a young female patient admitted for regional lymphadenitis (27). *B. quintana* is primarily transmitted through contamination of skin breaks by louse feces or crushed lice, with humans serving as its natural reservoir host, thus requiring no feline involvement. This pathogen chiefly causes trench fever and disseminated infections. Our case represents the first documented co-occurrence of active PTB and CSD-associated cervical lymphadenitis in an immunocompetent host, challenging the assumption that granulomatous lymphadenitis in TB-endemic regions is attributable to TB. Differentiating CSD from TB is challenging due to their similar histopathological features (necrotizing granulomas) and biases in epidemiological data. Notably, studies such as those by Gottlieb et al. and Machi et al. emphasize the misdiagnosis of CSD as TB lymphadenitis due to false-positive IGRA results or prevalence-based diagnostic presumptions (14, 15). Our case illustrates this challenge: the initial diagnosis relied on regional TB endemicity, positive IGRA results, and pulmonary CT findings, despite negative TB staining and TB DNA testing on lymph node specimens. In this instance, clinicians opted for MetaPath™ as a diagnostic approach for CSD. This contrasts sharply with the findings of Tasato et al. (18), where CSD was diagnosed only after empirical anti-TB treatment failed, underscoring the potential of advanced metagenomic diagnostics in preventing iatrogenic overtreatment.

**TABLE 1:** Summary of the characteristics of 15 cases diagnosed with cat scratch disease.

No.	References	Age (year)/sex	Chief complaints	Affected site	Cat contact (yes/no)	Granulomatous (yes/no)	Key findings/case features	CSD diagnosis method
1	Saito et al. (10)	79 F	Persistent fever, painful axillary lymphadenopathy	Axillary LN, liver, spleen, bone	Yes	Yes	Disseminated CSD, immunocompromised, no TB involvement	Pathological, serum antibody, PCR (LN)
2	Shariffudin et al. (11)	6 M	Blurred vision	Eye	Yes	No	Ocular bartonellosis (neuroretinitis), no LN/TB involvement	serum antibody
3	Machi (14)	61 F	Left inguinal mass	Inguinal lymphadenopathy	Yes	Yes	Inguinal involvement, CSD mimicking TB	Serum antibody, pathological
4	Gottlieb et al. (15)	36 F	Axillary mass	Axillary LN	Yes	Yes	CSD misdiagnosed as TB lymphadenitis, diagnosed by PCR	PCR (LN), pathological
5	Weinspach et al. (16)	5F,7M,10F,11F,13M	Varied (pain, fever, lymphadenopathy)	Varied (eye, LN, liver, spleen)	Yes	Yes	5 atypical pediatric cases, no TB involvement	Pathological, serum antibody
6	Matias et al. (17)	71 M	Axillary lymph node swelling	Axillary LN	Yes	Yes	CSD and pleural TB (diagnosed in 3 years after CSD treatment completion), malignancy background	Serum antibody, pathological, effective treatment for CSD
7	Tasato et al. (18)	74 F	Fever, cervical lymphadenopathy, general fatigue	Cervical LN	No	Yes	No cat exposure, history of TB, strongly positive TB IGRA, mimic TB	Serum antibody, pathological, PCR (LN), anti-TB drugs ineffective, clarithromycin effective
8	Mantis et al. (19)	44 F	Cough, whitish sputum production, painful enlarged neck LN	Generalized LN	Yes	Yes	CSD in AIDS, delayed diagnosis, mimicked TB	Pathological biopsy
9	Kagatani et al. (20)	23 M	19-day history of high fever	Hepatic	Yes	No	Atypical presentation, disseminated CSD (hepatic and bone involvement),WB-MRI	PCR (liver abscess aspirate), serum antibody, pathological biopsy (liver)
10	Doğan et al. (21)	30 F	Fever, arthralgia, night sweats, weight loss	Cervical, inguinal lymph nodes, liver, spleen	Yes	Yes	Suspected opportunistic CSD in hemodialysis, no TB details, immunocompromised host	Pathological, effective treatment for CSD
11	Li et al. (22)	37 F	Blurred vision	Fundus	Yes	No	Atypical presentation, *bartonella* neuroretinitis, mNGS, no TB involvement	Fundus examination, mNGS, serum antibody
12	Atıcı et al. (23)	12 M	Fever, abdominal pain, headache, weight loss	Inguinal lymphadenopathy, hepatic mass	Yes	Yes	Pediatric, atypical CSD (hepatic mass)	Serum antibody, pathological, PCR, effective treatment for CSD
13	Christensen et al. (24)	20 M	Night sweats, fluctuating fever	Axillary, iliac and inguinal lymph nodes, bones	Yes	Yes	Atypical CSD, severe osteomyelitis resulting from *Bartonella* infection, immunocompromised host	Serum antibody, PCR (LN), effective treatment for CSD
14	Waseem et al. (25)	23 M	Neck swelling, fever, generalized weakness, unconscious	Cervical LN	Yes	Yes	Typical CSD lymphadenitis, rule out TB	Cat exposure, pathology, effective treatment for CSD
15	Karski et al. (26)	1.5 F	Left arm movement restricted	Left arm	Yes	Yes	Pediatric CSD, suppurative LN, no TB involvement	Pathological, serum antibody

M, male; F, Female; TB, Tuberbculosis; LN, lymph node; MTB, Mycobacterium tuberculosis; CSD, Cat Scratch Disease; HIV, Human Immunodeficiency Virus; AIDs, Acquired Immune Deficiency Syndrome; IGRA, Interferon-Gamma Release Assay; mNGS, metagenomic Next Generation Sequenc.

Recognizing a history of cat exposure is crucial for diagnosing CSD, yet it is often overlooked. Notably, in 14 out of 15 analyzed cases, cat exposure was reported, although some cases lacked a clear history of scratches–similar to our patient, who was unable to recall any scratches or bites from a cat. This highlights the necessity of investigating even ambiguous instances of cat scratches or bites in suspected CSD cases, especially when lymph node samples yield negative results for specific stains (such as acid-fast and PAS) and molecular tests for MTB. Our case further illustrates that TB may coincidentally occur in patients with CSD, necessitating a comprehensive evaluation for dual pathogen infection. Immunocompromised hosts face higher risks of disseminated CSD (10, 19–21), however, our case demonstrates that dual infection can occur regardless of immune status. Among the 15 case studies reviewed, 9 documented atypical manifestations of CSD. Patients with compromised immunity, exhibit atypical manifestations, as the pathogen disseminates through lymphatic or bloodstream flow, leading to infections in organs and tissues beyond the lymph nodes. In these individuals, CSD can affect various systems, including the eyes, nervous system, musculoskeletal system, skin, breast, liver, spleen, and bones, and may also present with a range of other manifestations and syndromes, such as pneumonia, pleural effusion, erythema multiforme, hypercalcemia, glomerulonephritis, and myocarditis (10, 11, 16, 17, 19–24). The remaining 6 case reports predominantly documented fever or localized lymphadenopathy as primary presenting manifestations (14, 15, 17, 18, 25, 26). These cases further validate that the early phase of CSD infection typically begins with a papule or pustule at the site of inoculation, often accompanied by symptoms such as fever, fatigue, and nodular erythema. Concurrently, regional lymphadenopathy appears, usually unilateral, with about half of the cases involving a single lymph node, while the other half may progress to multiple lymph nodes. The disease can also lead to serious complications including cardiac issues (endocarditis), respiratory problems (pneumonia and pleural effusion), musculoskeletal conditions (osteomyelitis and paraspinal abscess), and central nervous system involvement (meningitis) (11, 16, 20, 22). Integrated with clinical features and ancillary investigations, mNGS provides a rapid and unbiased approach for detecting *Bartonella* species across diverse specimen types, particularly serving as a priority diagnostic tool for patients presenting with atypical manifestations (22).

The MetaPath™ platform (KingMed Center), an integrated diagnostic system that utilizes probe-based pathogen enrichment and mNGS technology, was employed for metagenomic pathogen detection from FFPE tissue sections to enhance pathogen detection rates through optimized sample processing and bioinformatics analysis. DNA was extracted following deparaffinization, enzymatic lysis, and purification, after which libraries were constructed through fragmentation, end repair, adapter ligation, and PCR amplification, followed by hybridization capture using biotinylated probes targeting over 3,000 pathogens. Sequencing was performed on an Illumina MiniSeq platform with single-end 100 bp reads, and bioinformatic analysis was conducted in the final step. A study conducted by Li M et al. demonstrated that the MetaPath™ platform achieved superior diagnostic performance for tuberculosis, with a sensitivity as high as 92.8%, significantly surpassing conventional TB detection methods such as PCR, acid-fast bacilli smear microscopy, and mycobacterial culture (28). This probe capture-based metagenomic sequencing approach addresses key limitations of traditional assays by efficiently suppressing human nucleic acid interference, tolerating ultra-low DNA inputs (<5 ng), providing rapid results, and enabling simultaneous identification of polymicrobial infections. At our tuberculosis-specialized hospital, infectious disease testing is limited to a narrow range of pathogens (29). Given that the MetaPath™ platform offers broad pathogen screening and demonstrates significant advantages for FFPE pathology specimens, and considering that the patient has such FFPE pathology samples available from an external hospital, the physician has opted to employ the MetaPath™ platform to test for the patient’s pathogens. The MetaPath™ platform played a pivotal diagnostic role in our co-infection case involving CSD-associated lymphadenitis and PTB, with the experimental methodology of the MetaPath™ platform detailed in the Supplementary file. At its core, the MetaPath™ platform comprises an optimized mNGS workflow. mNGS facilitates the simultaneous detection of tens of thousands of pathogens from limited samples, proving invaluable for diagnosing rare, atypical, and complex infections (30–32). A thorough search on PubMed using terms such as “metagenomic next-generation sequencing,” “cat scratch disease,” and “*Bartonella henselae*” revealed seven studies (see Table 2) that utilized mNGS on various sample types, including lymph node tissue, tissue swabs, intraocular fluid, plasma, peripheral blood, and cerebrospinal fluid, for diagnosing CSD (22, 31–36). Li et al. (31), Zhou et al. (32). reported mNGS diagnosing CSD lymphadenitis in TB-endemic regions where conventional methods yielded false negatives. Additionally, some studies by Hong et al. and Li et al. demonstrated the use of mNGS to confirm *B. henselae* in neuroretinitis cases that were initially negative through serological and PCR testing (22, 33). In cases of severe complications such as hemophagocytic lymphohistiocytosis or neurological issues, mNGS provided swift pathogen identification when standard tests were inconclusive (34, 35). Wang et al. further confirmed that mNGS exhibits greater sensitivity compared to culture methods in skin and soft tissue infections (36). Key advantages of mNGS include its unbiased detection of pathogens, the ability to identify difficult-to-culture organisms like *Bartonella*, and a rapid turnaround time of less than 48 h (22, 37). Compared to traditional PCR and serological antibody testing, MetaPath™ was chosen for its capability to simultaneously detect a wide array of known and unknown pathogens, its utilization of targeted probes to minimize host background noise, the supplementary genomic information it offers for pathogen characterization, and its compatibility with challenging sample types such as tissues and FFPE (28). Although mNGS technologies such as MetaPath™ offer significant advantages, but it’s limitations must be acknowledged. First, while probe-capture methods improve specificity, the high sensitivity of the method can lead to contamination, complicating the differentiation between colonizers, background microbes, and actual pathogens (38). Second, the associated costs are currently higher than those of conventional PCR or serology, which may restrict accessibility in resource-limited settings. Third, the requirement for specialized equipment and bioinformatics expertise further limits its widespread adoption in routine clinical practice. Thus, correlation with clinical presentation and traditional microbiological results remains essential. Additionally, as we are a pulmonary specialty hospital and do not offer serological testing for *Bartonella*, this patient did not undergo *Bartonella* IgM/IgG testing, which is a significant limitation of this case. Despite its limitations, mNGS is vital for diagnosing granulomatous lymphadenitis in areas where TB is common, as overlapping clinical features can lead to diagnostic delays. As evidenced by this case and previous literature, its early integration, particularly in patients with cat exposure, can prevent misdiagnosis, optimize therapy, and reduce iatrogenic harm.

**TABLE 2:** Summary of seven cases with the application of mNGS in diagnosis of cat scratch disease.

No.	References	Age (year)/sex	Contact with cats	Chief complaints	Sample type	*B. henselae*-specific sequences (reads)^a^	Relative abundance
1	Our study	56	Yes	Cervical masses	FFPE histopathological sections of a cervical lymph node specimen	82	7.19%
2	Li P et al. (22)	37 F	Yes	Blurred vision	Aqueous humor	521	2.26%
3	Li M et al. (31)	13 M	Yes	Intermittent fever, headache, poor appetite and weight loss	Peripheral blood	4	NA
4	Zhou T et al. (32)	51 M	Yes	10 days of fever with no obvious cause	Blood	3	100%
5	Hong H et al. (33)	11 M	Yes	Blurry vision persisting for 5 days	Vitreous fluid samples from the right eye	NA	0.71%
6	Yang T et al. (34)	48 F	Yes	Intermittent fever, systemic rash, fatigue, anorexia, weight loss, shock and unconsciousness	Lymph node tissue	7182	13.94%
7	Kassab I et al. (35)	49 M	No	Fever, tensionlike headache, nausea, and non-bloody emesis	Cerebrospinal fluid	22	NA
8	Wang Q et al. (36)	65 M	NA	Fever, subcutaneous abcess	Tissue swab	305	0.90%

^a^The number of reads specific to *Bartonella henselae* detected by metagenomic next-generation sequencing. M, male; F, Female; NA, not available; FFPE, formalin-fixed paraffin-embedded.

Although granulomatous cervical lymphadenitis in TB-endemic regions is frequently attributed to TB, this case presents the first reported co-occurrence of PTB and *B. henselae* associated lymphadenitis revealing critical diagnostic challenges. Histopathological overlap (granulomatous inflammation) and positive IGRA initially suggested tuberculous lymphadenitis. Crucially, MetaPath™ technology detection of *B. henselae* sequences in lymph node tissue –triggered by a history of cat exposure, even in the absence of clear scratches and negative results from special stains (PAS, silver methenamine, acid-fast) and TB-DNA–validated the diagnosis of CSD. This incidental discovery of PTB during the evaluation of cervical lymphadenopathy highlights how granulomatous inflammation may be misdiagnosed as TB without a history of cat exposure, particularly in areas with high TB prevalence.

## Conclusion

This case marks the first recorded occurrence of simultaneous active PTB and cervical lymphadenitis associated with CSD in a patient with a competent immune system. It underscores critical diagnostic challenges in TB-endemic regions, where granulomatous lymphadenitis is frequently misattributed to TB. The initial misdiagnosis of our patient highlights the histopathological similarities between TB and CSD, specifically the presence of necrotizing granulomas, as well as the limitations of relying solely on epidemiological context–such as TB-endemicity–and positive IGRA or PTB findings. A definitive diagnosis of CSD was established only after performing MetaPath™ technology on lymph node tissue, which was prompted by a history of cat contact, despite the absence of a clear cat scratch and negative results from traditional staining and TB-DNA tests. This incidental discovery of PTB during the evaluation of CSD emphasizes that granulomatous lymphadenitis may obscure non-tuberculous etiologies, particularly zoonotic infections. A comprehensive history, including cat exposure, and the early detection of *B. henselae* using advanced diagnostic techniques such as mNGS or MetaPath™ are essential for accurate diagnosis and appropriate treatment, thereby preventing potential iatrogenic harm and improving patient outcomes.

## Data Availability

The original contributions presented in this study are included in this article/Supplementary material, further inquiries can be directed to the corresponding author.
