# Supplementary material for: Coexistence of cat scratch disease lymphadenitis and active pulmonary tuberculosis in an immunocompetent host – a case report with metagenomic diagnosis and literature review
Source: Front Med (Lausanne). 2025 Oct 9;12:1667171. doi: 10.3389/fmed.2025.1667171 (PMC12544461; doi:10.3389/fmed.2025.1667171)
Supplement: Supplementary file 1 [file Table_1.docx]

**Metagenomic pathogen detection workflow (MetaPath™)**

**Sample processing and nucleic acid extraction**

DNA extraction from FFPE tissue sections (4 mm thick; 8 sections per sample) was performed as follows: Deparaffinization was carried out using a deparaffinization solution (56°C, 10 min, 400 rpm agitation), followed by centrifugation (15,000 rpm, 2 min). After supernatant removal, tissues were lysed in 750 mL ATL buffer supplemented with 20 mL proteinase K (56°C, overnight, 600 rpm orbital shaking). Lysates were homogenized with 250 mL of 0.5 mm glass beads (vortexing, 4000 rpm, 2 min), and DNA was purified using the MataPure™ DNA Extraction Kit (KS121-WSWTO-48) following the manufacturer’s operational manual and was used for the construction of libraries.

**Library construction, target enrichment, and sequencing**

Libraries were constructed through fragmentation, end repair, adapter ligation, and PCR amplification. Then, eight uniquely barcoded libraries were pooled for hybridization and captured using specific biotinylated probes for 2 hours after library generation using the MetaPath Pathogen Capture Metagenomic Assay Kit (KingCreate, China). The enrichment panel targeted >3,000 pathogens, including viruses, bacteria, fungi, and parasites. The pathogen target was screened according to the following rules: (I) the target had at least one completed genome sequence; (II) the target had at least one article related to human infection; (III) the target had research reports. Finally, this capture range was 1,850 bacterial, 692 fungal, 357 viral, and 226 parasitic species. Agilent 2100 was used for the quality control of the libraries and showed peaks near 350 bp. The Qubit dsDNA HS Assay Kit (Thermo Fisher Scientific Inc., Waltham, MA, USA) was used to construct quality-qualified libraries, and sequencing was performed on an Illumina MiniSeq platform set to 100-bp single end with average of 1 million reads per sample.

**Bioinformatic analysis**

Bioinformatic analyses of Metagenomic pathogen data was conducted according to the following process. Clean reads were obtained by removing sequencing adapters, reads of low quality, excessive N bases, or reads with length below 35 bp using fastp (version 0.23.1). The remaining reads were aligned to the human reference (GRCh38) using BurrowsWheeler aligner (BWA; version 0.7.17-r1188; Linux, San Francisco, CA, USA), and human reads were filtered. Subsequently, the reads were compared to the custom NCBI pathogen database (comprising 13,214 bacterial, 9,811 viral, 3,180 fungal, and 405 parasitic genomes) using an in-house pipeline. A pathogen was considered positive if (i) the RPM (reads per million total non-human reads) ≥10, (ii) the reads spanned≥3 non-overlapping genomic regions. RPM values were calculated with custom Python scripts (v3.9) and visualized in IGV (v2.15) for manual review of alignment quality.
